# LapDINO: A DINOv3 and Laplacian Pyramid-Based Approach for Outdoor Terrain Segmentation

**DOI:** 10.3390/s26123843

**Published:** 2026-06-17

**Authors:** Shiquan Ling, Xingchen Qin, Wenkang Xu, Mingmin Fu, Hao Huang, Shijie Ma, Zhenyu Liu

**Affiliations:** 1 School of Mechanical Engineering, Zhejiang University, Hangzhou 310030, China; 2 Geely Auto, Hangzhou 310051, China

**Keywords:** off-road terrain segmentation, DINOv3, laplacian pyramid, bidirectional cross-attention, multi-branch attention, visual adapter

## Abstract

As autonomous driving technology expands from structured urban roads to unstructured outdoor environments, precise understanding of complex terrain has become a critical requirement for ensuring safe vehicle navigation. However, outdoor environments are characterized by high dynamics, drastic illumination variations, ambiguous category boundaries, and prohibitive annotation costs, making traditional supervised learning methods that rely on large amounts of pixel-level annotations difficult to generalize. In this paper, we propose a novel dual-path bidirectional interactive encoder, termed LapDINO, that effectively combines the strong semantic generalization capability of the self-supervised foundation model DINOv3 with the multi-scale frequency analysis capacity of the Laplacian pyramid. Specifically, we leverage DINOv3 to extract global semantic features as a “semantic map”, while simultaneously obtaining multi-scale high-frequency details through Laplacian pyramid decomposition as “structural contours”. Building upon this, we design a bidirectional cross-attention fusion mechanism that enables dynamic interaction and mutual refinement between semantic information and geometric details. Furthermore, we introduce a multi-branch attention enhancement module that extracts pyramid features from three complementary perspectives. To address domain shift, we design lightweight visual adapters that enable efficient fine-tuning of the frozen DINOv3 backbone. Finally, we construct two off-road terrain segmentation datasets, VOTD and VOCD, to facilitate research in this domain. Experimental results demonstrate that the proposed method achieves state-of-the-art performance, striking an optimal balance between accuracy and computational efficiency, thereby providing a robust and efficient engineering solution for terrain perception in off-road environments.

## 1. Introduction

The rapid evolution of autonomous driving systems has shifted the focus from well-structured urban environments to complex, unstructured off-road terrains. In these wild scenarios, precise terrain perception is not merely a perceptual task but a critical safety prerequisite for navigation. Unlike city roads with clear lane markings and standardized signage, off-road environments are characterized by high dynamicity, drastic illumination variations, ambiguous class boundaries, and a lack of semantic regularity. These factors pose significant challenges to traditional computer vision pipelines. Conventional supervised learning approaches, which rely heavily on large-scale pixel-level annotations, struggle to generalize in such settings due to the prohibitive cost of data labeling and the inherent domain gap between training datasets and the complex diversity of real-world terrains.

Recent advances in self-supervised learning have offered a promising alternative by leveraging vast amounts of unlabeled data to learn robust visual representations. Notably, the emergence of foundation models like DINOv3 [1] have demonstrated remarkable capabilities in capturing global semantic priors without manual supervision. However, directly applying these general-purpose models to off-road terrain segmentation reveals critical limitations. While DINOv3 excels at recognizing high-level semantic concepts, its features often lack the high-frequency geometric details necessary to delineate fine-grained terrain structures. Conversely, traditional multi-scale methods like Laplacian pyramids are adept at capturing frequency-specific details but lack the semantic reasoning ability to distinguish between visually similar yet semantically distinct terrains (e.g., mud and dirt).

**Figure 1:**
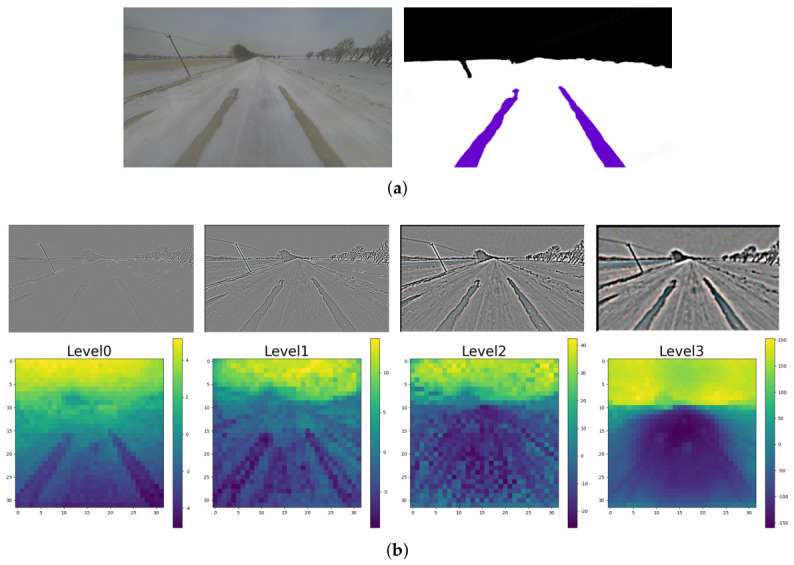
Complementary feature representations. The Laplacian pyramid preserves multi-scale geometric details from high-frequency textures to structural contours, while DINOv3 captures hierarchical semantic information from local patterns to global context. (**a**) Input image and corresponding Ground Truth mask. (**b**) Multi-level feature visualization. Upper: Laplacian pyramid features from fine to coarse ($L_{0}$ to $L_{3}$). Lower: PCA projections of DINOv3 features from shallow to deep layers (2, 5, 8, 11).

To illustrate the complementary nature of the two representations, Figure 1 visualizes features extracted by the Laplacian pyramid and DINOv3 at multiple levels. The Laplacian pyramid progressively captures geometric details from fine textures to coarse structural contours, preserving high-frequency information often lost in deep networks. In contrast, DINOv3 encodes hierarchical semantics: shallow layers respond to local edges and textures, while deeper layers aggregate global context for terrain discrimination. This complementarity between geometric fidelity and semantic abstraction motivates our bidirectional fusion mechanism, where semantic context guides detail enhancement while geometric features refine boundary localization.

To bridge the gap between semantic abstraction and geometric precision, we propose LapDINO, a dual-path framework that synergistically fuses global context with local structural details. Unlike cascaded approaches, LapDINO integrates DINOv3’s semantic generalization with Laplacian pyramid multi-scale analysis through a unified architecture featuring three key innovations. First, a Bidirectional Cross-Attention Fusion mechanism dynamically refines ambiguous structures via semantic priors while correcting semantic hallucinations using high-frequency geometric cues. Second, a Multi-Branch Attention module enhances feature representation across channel, spatial, and content dimensions to preserve critical traversal cues. Finally, a lightweight visual adapter enables parameter-efficient fine-tuning for the off-road domain without catastrophic forgetting. This cohesive design ensures robust terrain perception by harmonizing heterogeneous semantic and geometric representations.

The contributions of this work are summarized as follows:

- We propose LapDINO, a novel framework to synergistically combine self-supervised foundation models with multi-scale frequency analysis for off-road terrain segmentation, effectively balancing semantic robustness and geometric fidelity.
- We design a Bidirectional Cross-Attention Fusion mechanism and a Multi-Branch Attention Enhancement module to enable deep, dynamic interaction between global semantics and local structural details, significantly improving segmentation accuracy at complex boundaries.
- We introduce a lightweight visual adapter strategy that allows for efficient domain adaptation of frozen foundation models, reducing computational overhead while maintaining state-of-the-art performance.
- We construct and release two comprehensive off-road terrain segmentation datasets, VOTD and VOCD, to facilitate future research in unstructured environment perception.
- Experiments demonstrate that our method achieves state-of-the-art results on multiple benchmarks, offering a robust and efficient solution for autonomous navigation in challenging wild environments.

## 2. Related Work

### 2.1. Semantic Segmentation for Terrain Recognition

Terrain perception is essential for intelligent off-road systems, with methods evolving from traditional approaches based on geometry and texture, to CNN-based semantic segmentation, and now to Transformer-based methods. Early works [2,3] extracted terrain features using color and texture with nearest neighbor classifiers. Refs. [4,5] utilized Speeded-Up Robust Features (SURFs) [6] to obtain discriminative descriptors of the ground, offering better robustness to noise, and trained Support Vector Machines (SVMs) [7] or Multi-Layer Perceptrons as classifiers. These traditional methods rely heavily on hand-crafted features and classifiers, achieving reasonable accuracy in controlled settings but exhibiting limited generalization under varying illumination, seasons, and terrain diversity.

Deep learning methods using CNNs and Transformers have become mainstream for terrain semantic segmentation. Ref. [8] applied the Fully Convolutional Network [9] to terrain semantic segmentation, categorizing terrain into Sky, Road, Grass, and Vegetation, while the TU-net [10] based on U-Net [11] improved accuracy with RGB-only inputs for terrain classification. The work in [12] generated segmentation labels using DeepLabv3+ [13] for Martian terrain assessment. Subsequent works combined CNNs with traditional color clustering [14], employed group-wise attention to segment terrains for robot navigation [15], compared CNN models for terrain classification [16], designed a multi-scale CNN-based encoder–decoder [17], used ResNet34 and U-Net architectures [18], and incorporated uncertainty estimation modules [19]. However, these traditional approaches hinder their generalization capability in practical intelligent driving applications.

### 2.2. Vision Foundation Models

Recently, Vision Foundation Models (VFMs) have emerged as a promising solution to these limitations. Meta’s Segment Anything Model (SAM) [20] and its successor SAM2 [21], trained on over a billion masks, demonstrate remarkable zero-shot segmentation capabilities and generalization. However, SAM is fundamentally designed for prompt-driven instance segmentation and lacks inherent semantic understanding, making it unsuitable for real-time, dense semantic prediction required in terrain segmentation.

In contrast, self-supervised models such as DINO [22], DINOv2 [23], and DINOv3 [1] learn rich, general, and disentangled visual representations from massive unlabeled image collections. DINOv3 features have shown strong performance across diverse downstream tasks, offering exceptional feature representation and cross-domain generalization. A key challenge and the focus of this work is how to effectively adapt and transfer the powerful abilities of these foundation models to the specific domain of terrain segmentation.

### 2.3. Multi-Scale Pyramidal Feature Representation

Multi-scale feature representation is essential for computer vision tasks that require both global context and local detail. The Laplacian pyramid, introduced by [24], decomposes an image into multiple frequency bands via recursive Gaussian blurring and subtraction, separating low-frequency and high-frequency components. This is especially useful in terrain segmentation, where accurate boundaries and scene-level understanding are both critical.

In semantic segmentation, ref. [25] proposed Laplacian Pyramid Reconstruction and Refinement, which refines coarse predictions by progressively adding high-frequency details through multiplicative gating. Other works [26,27,28] apply Laplacian pyramids to cross-scale feature fusion for low-light image enhancement and dark object detection. These studies show that explicitly modeling hierarchical frequency bands yields robust features under challenging illumination and environmental conditions.

In contrast, we leverage the Laplacian pyramid to capture discriminative terrain features across scales and fuse them with DINOv3’s self-supervised representations. By combining classical multi-scale decomposition with modern foundation models, our approach significantly improves terrain segmentation robustness in diverse outdoor settings.

### 2.4. Outdoor Terrain Segmentation Datasets

Datasets are fundamental to advancing terrain perception research. Compared to highly standardized urban driving datasets, outdoor terrain datasets face significant challenges, including high annotation costs, environmental variability, and complex category definitions. For structured roads, datasets such as CamVid [29], Cityscapes [30], KITTI [31], and SemanticKITTI [32] are widely used in autonomous driving research. However, in these datasets, terrain is predominantly paved and often labeled simply as “road”, lacking the fine-grained terrain categorization required for outdoor navigation.

In contrast, datasets for unstructured outdoor environments are central to terrain segmentation research. RUGD [33] is a dataset dedicated to off-road semantic segmentation, collected by a small unmanned ground vehicle across diverse natural and semi-urban environments, providing a benchmark for evaluating off-road perception algorithms. RELLIS-3D [34] is a multimodal off-road dataset featuring synchronized LiDAR scans and annotated images, captured at Texas A&M University’s Rellis Campus, and presents challenges related to class imbalance and environmental variability. GOOSE [35] is a large-scale, multi-season, multi-weather off-road dataset that includes pixel-level annotated RGB images and LiDAR point clouds, covering a wide range of common terrain classes in unstructured outdoor settings. However, these datasets suffer from incomplete terrain category coverage: RUGD and RELLIS-3D lack snow, while GOOSE lacks mud. To address these limitations, we merge these three datasets through category mapping to create the VOCD dataset with 7 common terrain categories. Additionally, we introduce VOTD dataset, collected under real-vehicle driving conditions, which includes 11 detailed terrain categories.

## 3. Method

### 3.1. Overview

**Figure 2:**
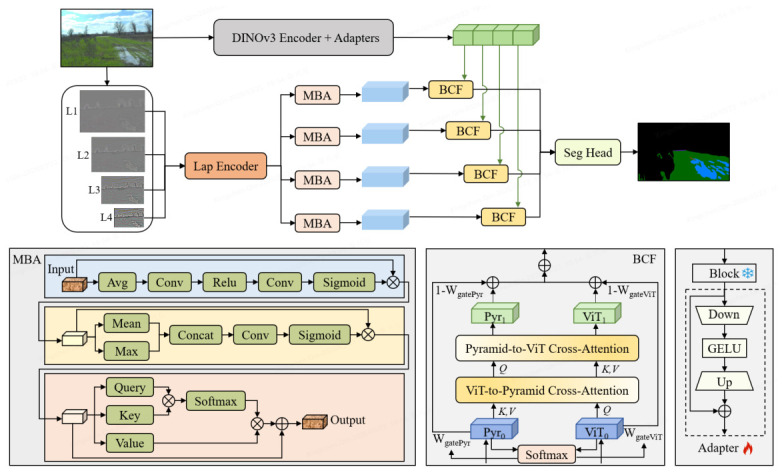
Overview of the proposed LapDINO. The overall architecture consists of four key components: (1) a frozen DINOv3 visual encoder enhanced with lightweight visual adapters for domain adaptation, (2) a Laplacian pyramid geometric encoder with multi-branch attention enhancement, (3) a bidirectional cross-attention fusion module that enables mutual guidance between semantic and geometric features, and (4) a progressive decoder for high-resolution segmentation.

We present LapDINO, a novel dual-path framework for outdoor terrain semantic segmentation that synergistically combines the semantic richness of self-supervised DINOv3 with the multi-scale structural details captured by Laplacian pyramid decomposition. The overall structure of LapDINO is shown in Figure 2.

Given an input image $I\in R^{3\times H\times W}$, LapDINO extracts complementary feature representations through two parallel pathways. The semantic pathway leverages DINOv3 equipped with Adapters to capture global contextual understanding and high-level semantics, while the geometric pathway employs Laplacian pyramid decomposition to capture multi-scale texture details and structural boundaries. The resulting Laplacian pyramid features at different levels are then processed by a hierarchical Lap Encoder, which progressively downsamples each level to achieve spatial alignment with the DINOv3 feature resolution. These spatially aligned geometric features are subsequently refined through a Multi-Branch Attention (MBA) module, which enhances discriminative texture information from channel, spatial, and content perspectives. The enhanced geometric features, together with the DINOv3 semantic features, are then fed into a bidirectional cross-attention fusion (BCF) mechanism, enabling semantic-guided detail enhancement and detail-refined boundary correction. Finally, the fused multi-scale features from all pyramid levels are aggregated and progressively upsampled to produce pixel-wise segmentation predictions.

### 3.2. DINOv3 Encoder and Adapters

We employ DINOv3-S/16 as our semantic backbone, pre-trained on massive unlabeled images through self-distillation. The backbone parameters remain frozen during training to preserve the rich semantic knowledge learned from large-scale pre-training. Given an input image $I\in R^{3\times 512\times 512}$, we first partition it into 16×16 patches, and the DINOv3 encoder processes these patches through 12 transformer blocks. To capture multi-layer semantic information, we extract intermediate features from layers {2,5,8,11}, obtaining $F_{l}^{\mathrm{map}}\in R^{B\times D\times 32\times 32}$ for each layer *l*. These four feature maps, corresponding to shallow to deep layers, are then concatenated as $F_{s}^{\mathrm{concat}}=\mathrm{Concat}\left({\left\{F_{l}^{\mathrm{map}}\right\}}_{l}\right)\in R^{B\times 4D\times 32\times 32}$. A final 1×1 convolution fuses the multi-layer features into a compact semantic representation $F_{s}={\mathrm{Conv}}_{1\times 1}\left(F_{s}^{\mathrm{concat}}\right)\in R^{B\times D\times 32\times 32}$. Through this concatenation and fusion, the resulting semantic feature $F_{s}$ aggregates complementary information across different levels, integrating fine-grained details from shallow layers and high-level semantic context from deep layers, thereby providing a comprehensive representation for terrain understanding.

Directly applying DINOv3 to terrain images suffers from domain shift due to distribution differences between training images and off-road scenes. Full fine-tuning would require extensive labeled data and risks catastrophic forgetting of pre-trained knowledge. We therefore introduce lightweight visual adapters that enable efficient domain adaptation while keeping the backbone frozen. We insert adapters after each multi-head self-attention and feed-forward network block. An adapter is defined as $\mathrm{Adapter}\left(x\right)=x+\sigma \left(xW_{\mathrm{down}}\right)W_{\mathrm{up}}$, where $x\in R^{B\times N\times D}$ is the block output, $W_{\mathrm{down}}\in R^{D\times r}$ and $W_{\mathrm{up}}\in R^{r\times D}$ are learnable projection matrices with bottleneck dimension r=64, and σ(·) is the GELU activation. The residual connection ensures that the adapter initializes as identity mapping, allowing the model to preserve pre-trained knowledge while gradually adapting to terrain data during training.

### 3.3. Laplacian Pyramid Feature Extraction and Enhancement

Conventional convolutional and transformer architectures progressively downsample feature maps to capture semantic context, inevitably discarding high-frequency details such as fine textures, sharp edges, and subtle surface patterns that are essential for distinguishing visually similar terrain types. To explicitly preserve and enhance these geometric cues, we introduce a Laplacian pyramid decomposition mechanism that separates the input image into multiple frequency bands, enabling the network to retain and process structural details at varying scales.

Given an input image $I\in R^{3\times H\times W}$, we construct a *K*-level Gaussian pyramid $\left\{G_{0},G_{1},\ldots ,G_{K}\right\}$ and each subsequent level is obtained by applying Gaussian smoothing followed by 2× downsampling:

(1) $$G_{k+1}=\mathrm{Down}\left({\mathrm{Conv}}_{\mathrm{gauss}}\left(G_{k}\right)\right),\;k=0,\ldots ,K-1$$

The Laplacian pyramid captures the high-frequency details lost during downsampling as the difference between successive Gaussian levels:

(2) $$L_{k}=G_{k}-\mathrm{Up}\left(G_{k+1}\right),\;k=0,\ldots ,K-1$$

where Up(·) denotes bilinear upsampling followed by Gaussian smoothing. The final level $L_{K}=G_{K}$ represents the low-frequency residual. This decomposition yields a multi-scale representation $P=\{L_{0},L_{1},\ldots ,L_{K}\}$ where finer levels capture high-frequency textures and coarser levels encode structural contours.

To align these multi-scale geometric features with the semantic feature resolution, we apply a hierarchical encoder $E_{k}$ to each Laplacian level, producing a set of spatially aligned geometric features:

(3) $$F_{k}^{\mathrm{pyr}}=E_{k}\left(L_{k}\right)\in R^{B\times D\times h\times w},\;k=0,\ldots ,K-1$$

where h=H/16 and w=W/16 matching the DINOv3 feature resolution. Each encoder consists of stacked stride-2 convolutions, with deeper levels using fewer downsampling steps to preserve structural integrity.

To further enhance the discriminative capability of these geometric features, we introduce a multi-branch attention (MBA) module that sequentially applies three complementary attention mechanisms. The channel attention branch adaptively recalibrates feature importance across channels by squeezing spatial information through global average pooling and learning channel-wise weights via a bottleneck structure. The spatial attention branch generates attention maps by aggregating channel information through average and max pooling, followed by a 7×7 convolution:

(4) $$M_{\mathrm{spatial}}=\sigma \left({\mathrm{Conv}}_{7\times 7}\left([\mathrm{AvgPool}(F);\mathrm{MaxPool}(F)]\right)\right)$$

where σ denotes the sigmoid function. The content attention branch employs self-attention to model long-range dependencies across the feature map:

(5) $$\mathrm{Attn}\left(F\right)=\mathrm{Softmax}\left(\frac{QK^{⊤}}{\sqrt{d_{k}}}\right)V$$

where *Q*, *K*, and *V* are obtained via 1×1 convolutions. These three attention mechanisms operate in cascade, progressively refining the geometric features to emphasize discriminative textures while maintaining structural consistency.

After encoding and enhancement, we obtain a set of multi-scale geometric features $F_{\mathrm{pyr}}=\left\{F_{0}^{\mathrm{pyr}},F_{1}^{\mathrm{pyr}},\ldots ,F_{K}^{\mathrm{pyr}}\right\}$, each enriched with structured details at a specific scale. This multi-scale representation provides complementary information to the semantic features, enabling fine-grained texture understanding while preserving the global structural context essential for accurate terrain segmentation.

### 3.4. Bidirectional Cross-Attention Fusion

To fully exploit the complementarity between semantic and geometric representations, we propose a Bidirectional Cross-Attention Fusion (BCF) module that enables dynamic interaction between DINOv3 semantic features and Laplacian pyramid geometric features, rather than combining them through simple operations such as concatenation or addition. For each pyramid level *k*, let $F_{\mathrm{vit}}\in R^{B\times D\times h\times w}$ denote the semantic feature and $F_{k}^{\mathrm{pyr}}\in R^{B\times D\times h\times w}$ denote the geometric feature at that scale. The module executes two parallel cross-attention processes that operate in complementary directions.

In the semantic-to-geometry guidance branch, semantic features serve as queries to attend to geometric features. This allows global semantic context to selectively highlight high-frequency details that are relevant to the current terrain category while suppressing irrelevant noise such as shadows or sensor artifacts:

(6) $${\tilde{F}}_{k}^{\mathrm{pyr}}=\mathrm{CrossAttn}\left(Q=F_{\mathrm{vit}},\;K=F_{k}^{\mathrm{pyr}},\;V=F_{k}^{\mathrm{pyr}}\right)$$

Conversely, the geometry-to-semantic guidance branch uses geometric features as queries to attend to semantic features. This enables fine-grained structural details to refine semantic boundaries, injecting precise localization information into high-level representations:

(7) $${\tilde{F}}_{\mathrm{vit}}=\mathrm{CrossAttn}\left(Q=F_{k}^{\mathrm{pyr}},\;K=F_{\mathrm{vit}},\;V=F_{\mathrm{vit}}\right)$$

To dynamically balance the contributions of original and guided features, we introduce a learnable gated fusion mechanism. The gate weights are generated by concatenating the semantic and geometric features, followed by a 1×1 convolution that produces two-channel spatial attention maps normalized via softmax:

(8) $$\left[\alpha ,\;\beta \right]=\mathrm{Softmax}\left({\mathrm{Conv}}_{1\times 1}\left([F_{\mathrm{vit}},\;F_{k}^{\mathrm{pyr}}]\right)\right)$$

where $\alpha ,\beta \in R^{B\times 1\times h\times w}$ are spatial attention maps that sum to one. The weighted fusion is then performed independently for each modality:

(9) $$\begin{array}{cc} F_{\mathrm{vit}}^{\mathrm{fused}} & =\alpha ⊙{\tilde{F}}_{\mathrm{vit}}+\left(1-\alpha \right)⊙F_{\mathrm{vit}}\\ F_{\mathrm{pyr}}^{\mathrm{fused}} & =\beta ⊙{\tilde{F}}_{k}^{\mathrm{pyr}}+\left(1-\beta \right)⊙F_{k}^{\mathrm{pyr}} \end{array}$$

Finally, the fused features from both modalities are combined through element-wise addition to produce the output for scale *k*:

(10) $$F_{\mathrm{final}}^{\left(k\right)}=F_{\mathrm{vit}}^{\mathrm{fused}}+F_{\mathrm{pyr}}^{\mathrm{fused}}$$

This additive fusion preserves the complementary strengths of both representations while maintaining computational efficiency. The gate mechanism allows the network to adaptively decide, for each spatial location, whether to rely more on semantic priors or geometric details based on the scene context. After obtaining fused features from all pyramid levels $\left\{F_{\mathrm{final}}^{\left(0\right)},F_{\mathrm{final}}^{\left(1\right)},\ldots ,F_{\mathrm{final}}^{\left(K\right)}\right\}$, we concatenate them along the channel dimension and apply a linear projection layer for channel compression and integration, yielding the final multi-modal fused feature $F_{\mathrm{final}}$.

### 3.5. Decoder and Loss Function

To recover the segmentation mask to the original resolution from the fused feature $F_{final}\in R^{B\times D\times h\times w}$, we employ a lightweight fully convolutional decoder. This decoder consists of two 3×3 convolutional blocks to progressively refine features, followed by bilinear interpolation upsampling by a factor of 16. Finally, a 1×1 convolution maps the channels to the number of classes $C_{cls}$.

To address the severe class imbalance, we employ a composite loss function combining Focal Loss and Dice Loss. The Focal Loss focuses training on hard-to-classify pixels by down-weighting easy examples, while the Dice Loss directly optimizes spatial overlap to enhance the segmentation. The total loss is formulated as:

(11) $$L_{\mathrm{total}}=L_{\mathrm{focal}}+L_{\mathrm{dice}}$$

## 4. Experiments

### 4.1. Datasets

We conduct comprehensive experiments on two datasets: the proposed VOTD (A Vehicle Outdoor Terrain Driving Dataset) and the combined VOCD (A Vehicle Outdoor Combined Dataset). VOCD integrates three publicly available off-road datasets: RUGD [33], RELLIS-3D [34], and GOOSE [35], to address three critical limitations inherent in existing benchmarks. First, to achieve category completeness, we combine complementary classes that are missing from individual datasets: snow appears exclusively in GOOSE, while mud is absent in GOOSE but present in RUGD and RELLIS-3D. Second, to enhance environmental diversity, we aggregate samples spanning multiple seasons, diverse weather conditions, various geographic locations, and different acquisition setups. Third, to resolve inconsistent category definitions across datasets, we unify all classes into a standardized taxonomy of 7 vehicle driving terrain classes, producing a consolidated dataset with consistent annotations suitable for robust model training and evaluation.

VOTD is a newly collected dataset designed to address the scarcity of high-quality off-road terrain data captured from vehicle perspectives. Data acquisition was performed using a surround-view camera system mounted on an SUV, with images collected across diverse terrains. The dataset encompasses varied environmental conditions: multiple seasons, different weather scenarios, and diverse geographic locations. A total of 3886 distortion-corrected RGB images at resolution 1920×1080 were sampled at 1 frame per second from continuous video sequences, ensuring temporal diversity while maintaining manageable dataset size. The dataset comprises 11 fine-grained categories, enabling detailed terrain analysis for both research and practical applications.

**Figure 3:**
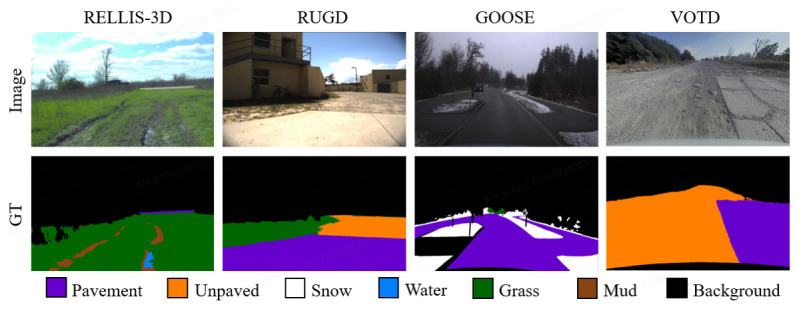
Visualization of images from each dataset and their corresponding mask GT.

Both datasets follow an 8:1:1 training, validation, and testing split, maintaining consistent class distributions across splits to facilitate fair evaluation. Detailed dataset statistics are presented in Table 1. The unified category mapping from original dataset labels to the standardized 7-class taxonomy is provided in Table 2. Representative visual samples from each dataset are shown in Figure 3.

### 4.2. Metrics

To comprehensively evaluate the performance of the proposed method on the terrain semantic segmentation task, we adopted the widely used evaluation metrics: Mean Intersection over Union (Mean IoU). Mean IoU measures the overlap between predicted and ground truth segmentation maps for each class:

(12) $$\mathrm{mIoU}=\frac{1}{C}\sum_{c=1}^{C}\frac{{\mathrm{TP}}_{c}}{{\mathrm{TP}}_{c}+{\mathrm{FP}}_{c}+{\mathrm{FN}}_{c}}$$

### 4.3. Implementation Details

During training, we employed the DINOv3-S/16 ViT [1] as the backbone and kept the pre-trained weights of the ViT frozen throughout. Input images were resized to 512 × 512. The batch size was set to 8, training was run for 50 epochs, with a learning rate of 0.001. AdamW [36] was chosen as the optimizer with a weight decay coefficient of $5\times 10^{-4}$. Data augmentation included random horizontal flipping with a probability of 0.5. Training was conducted on an NVIDIA RTX 3090 GPU with 24 GB of memory.

### 4.4. Comparison with State-of-the-Art Methods

To validate the performance advantages of our method on the terrain semantic segmentation task, we conducted systematic comparisons with current mainstream CNN-based and Transformer-based methods. All methods are trained and evaluated under the same hardware configuration and training strategy to ensure fairness. The VOCD and VOTD datasets represent different terrain environments and data distribution variations, offering diverse scenes and challenges, enabling evaluation of method generalization and robustness.

**Figure 4:**
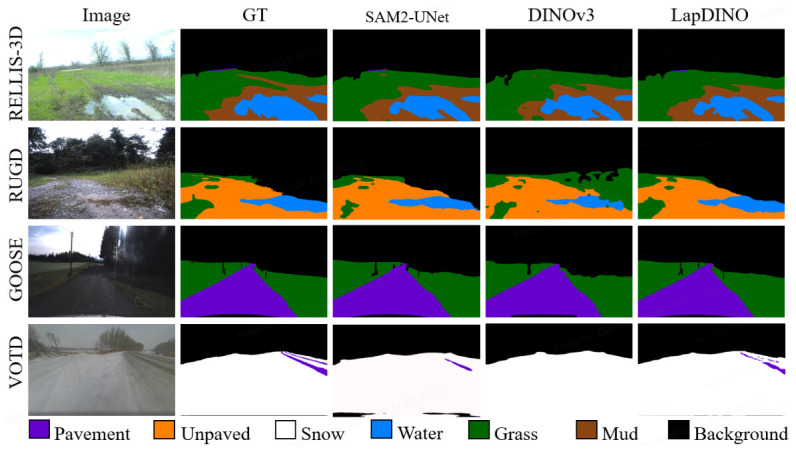
Qualitative comparison of the proposed method and others on different datasets. Our method shows better detail preservation.

Table 3 reports the backbone architecture, model size, computational cost, and segmentation performance of each method under both in-domain and cross-domain evaluation settings. All results are averaged over three independent training runs with different random seeds, and standard deviations are reported to reflect result stability. Notably, our LapDINO achieves not only the highest in-domain accuracy but also the best cross-domain generalization, demonstrating robust adaptability to unseen off-road environments. Importantly, LapDINO strikes an optimal balance between segmentation accuracy and computational efficiency, making it well-suited for real-world intelligent perception systems. Figure 4 shows that our method has better prediction details.

Beyond overall metrics, we further analyze per-class segmentation performance on the VOCD dataset to provide a granular understanding of each method’s strengths and weaknesses across different terrain types. As shown in Table 4, LapDINO consistently outperforms all competing methods across all terrain categories.

### 4.5. Ablation Study

To systematically evaluate the contribution of each component in the proposed LapDINO framework, we conduct ablation studies on the combined public VOCD dataset. We analyze four key modules: the DINOv3 visual adapters (Adapters) for domain adaptation, the Laplacian Pyramid Encoder (LPE) for multi-scale geometric feature extraction, the multi-branch attention module (MBA) for geometric feature enhancement, and the bidirectional cross-attention fusion module (BCF) for semantic–geometric interaction. For configurations without BCF, the Laplacian features and DINOv3 features are directly added element-wise after spatial alignment, serving as a baseline fusion strategy. The quantitative results are reported in Table 5.

Starting from a frozen DINOv3 baseline (75.43% mIoU), introducing either DINOv3 Adapters or the Laplacian Pyramid Encoder (LPE) yields significant improvements of +2.52% and +3.30%, respectively, validating the effectiveness of domain adaptation and multi-scale geometric modeling. Building upon LPE, the integration of the Multi-Branch Attention (MBA) module further boosts performance to 79.24% by enhancing feature representations across different dimensions within the Laplacian hierarchy. Crucially, we assess the impact of our fusion strategy: comparing Row 4 and Row 5 reveals that incorporating the Bidirectional Cross-attention Fusion (BCF) module elevates the mIoU to 80.15%, demonstrating that structured cross-modal interaction significantly outperforms naive feature aggregation. Finally, the full model, which synergizes all components, achieves the state-of-the-art performance of 81.57% mIoU. This final gain of +1.42% confirms that the collaboration between task-specific adapters and geometry-aware bidirectional fusion is essential for maximizing the complementarity of semantic and structural features.

We further investigate the impact of the adapter bottleneck dimension *r* on model performance and efficiency. As shown in Table 6, reducing *r* from 128 to 8 consistently improves segmentation accuracy, with r=8 achieving the highest mIoU. However, this improvement comes at a substantial cost in terms of parameter efficiency: the parameter increment rises from 0.03M to 0.46M as *r* decreases from 128 to 8, and the computational cost increases correspondingly from 0.03 to 0.42 GFLOPs. The marginal gain from r=64 to r=8 is only 0.51% mIoU, yet it requires approximately 7.7 times more additional parameters. Considering the practical requirements of embedded deployment on automotive-grade platforms, where both memory footprint and inference latency are critical constraints, we select r=64 as the optimal configuration. This choice delivers a substantial mIoU improvement of +1.42% over the no-adapter baseline while maintaining minimal parameter overhead and computational cost increase, striking an effective balance between accuracy and efficiency for real-world off-road terrain segmentation.

### 4.6. Deployment and Outdoor Testing

To evaluate the inference performance of the proposed method in real-world environments, we conducted outdoor vehicle tests covering all terrain classes under various seasons and weather conditions. LapDINO was deployed on the Huashan A1000 platform, an automotive-grade autonomous driving computing chip with a computing power of 58 TOPS (INT8). The model was integrated into the vehicle’s perception system and evaluated for real-time performance under actual driving scenarios.

Specifically, the original 1920×1080 RGB input frames from the vehicle-mounted camera were resized to 512×512 pixels using bilinear interpolation to match the input resolution expected by the model. We applied post-training INT8 quantization using a calibration dataset comprising 1000 representative frames sampled from our validation set under diverse illumination and weather conditions. The runtime system leveraged the Huashan A1000’s dedicated neural processing unit (NPU) via vendor-provided SDKs, with inference executed in a single-stage pipeline. Preprocessing and postprocessing were offloaded to the CPU cores integrated within the SoC, while the NPU handled all neural network computations. Under this configuration, the system achieved an average end-to-end latency of 42 ms per frame (including preprocessing, inference, and postprocessing), corresponding to a sustained throughput of approximately 24 FPS. This meets the real-time requirement for autonomous driving applications operating.

**Figure 5:**
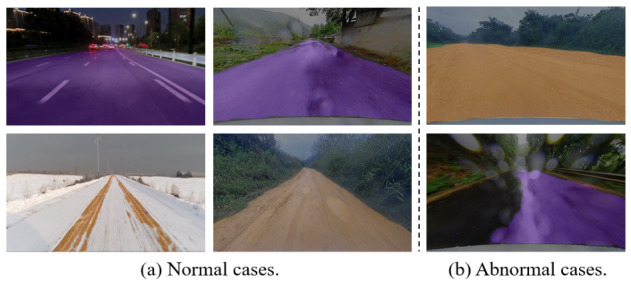
Qualitative results of LapDINO on outdoor vehicle testing. (**a**) Normal cases: robust segmentation under low-light, rain, snow, and mud. (**b**) Abnormal cases: failure modes including misclassification of mud (without puddles or tracks) as dirt road due to textural similarity, and partial missed detection under heavy rain with motion blur.

Figure 5 presents representative qualitative results from our outdoor vehicle tests, illustrating both the strengths and limitations of LapDINO under diverse real-world conditions. The normal cases (a) include low-light nighttime scenes, rain-affected roads, mixed snow-covered terrain, and muddy surfaces. However, as shown in the abnormal cases (b), challenges remain: for instance, muddy terrain lacking visible puddles or vehicle tracks is occasionally misclassified as unpaved dirt road due to the high textural similarity between smooth mud and dirt surfaces. Additionally, heavy rain combined with camera motion blur can lead to false positives or missed detections in traversable regions. These failure cases highlight that adverse weather conditions and motion artifacts are critical bottlenecks for current vision-based terrain segmentation systems, motivating future work on temporal fusion and multi-modal sensor integration to mitigate such errors.

## 5. Conclusions

In this paper, we have presented LapDINO, a novel dual-path framework for off-road terrain semantic segmentation that synergistically combines the semantic richness of DINOv3 with the multi-scale structural details captured by Laplacian pyramid decomposition. Through bidirectional cross attention fusion, semantic context guides detail enhancement while geometric boundaries refine semantic localization, effectively bridging the semantic–geometric gap in terrain understanding. Experiments demonstrate that LapDINO achieves state-of-the-art performance while maintaining computational efficiency suitable for real-time deployment. Despite these promising results, the current framework relies solely on single-frame RGB images and exhibits performance degradation under extreme weather conditions such as heavy rain, while also struggling with visually ambiguous surfaces where different terrain types share similar textures. Future work will focus on incorporating temporal information through video input to leverage motion cues, integrating stereo vision depth estimation to provide appearance-invariant geometric cues, and developing a unified framework that jointly learns terrain segmentation and vehicle–terrain interaction dynamics for more informed path planning.

## Figures and Tables

**Table 1:** Detailed information of datasets for terrain segmentation.

Dataset	Images	Resolution	Classes
RELLIS-3D	6235	1920 × 1200	20
RUGD	7453	1376 × 1110	24
GOOSE	8790	2048 × 1000	64
VOTD	3886	1920 × 1080	11

**Table 2:** Category mapping relationship for merging datasets. We unify the semantic categories from four datasets into common terrain categories relevant to vehicle outdoor driving scenarios. Terrain categories with similar visual appearance or driving characteristics are merged accordingly. Entries marked with “/” indicate categories not present in the original datasets.

Category	RELLIS-3D	RUGD	GOOSE	VOTD
pavement	asphalt, concrete	asphalt, concrete	asphalt, road_marking, pedestrian_crossing	asphalt, concrete
unpaved	dirt, rubble	dirt, sand, gravel, mulch, rock, rock-bed	soil, gravel, cobble, leaves	dirt, gravel, sand, mulch
snow	/	/	snow	snow
water	water, puddle	water	water	water
grass	grass	grass	low_grass, high_grass	grass
mud	mud	/	/	mud
background	tree, pole, vehicle, sky, object, building, log, person, fence, bush, barrier	tree, pole, vehicle, sky, container/generic-object, building, log, bicycle, person, fence, bush, sign, bridge, picnic-table	sky, construction, vehicle, road, object, sign, human, animal, vegetation (excluding grass)	/
void	void	void	undefined	void

**Table 3:** Complexity and performance comparison of different methods. LapDINO maintains competitive Params and GFLOPs while achieving state-of-the-art mIoU across both datasets. Here, *C* and *T* denote the VOCD and VOTD datasets, respectively. *C→T* indicates training on VOCD and evaluating on VOTD, while *T→C* indicates training on VOTD and evaluating on VOCD, demonstrating cross-dataset generalization capability. All mIoU values are reported as mean ± standard deviation over three independent training runs.

Method	Backbone	Params (M)	GFLOPs	mIoU (C)	mIoU (T)	mIoU (C→T)	mIoU (T→C)
UNet [11]	ResNet-50	41.9	92.2	78.5 ± 0.2	92.6 ± 0.3	50.3 ± 0.5	47.5 ± 0.5
PSPNet [37]	ResNet-50	44.5	59.2	78.9 ± 0.2	93.0 ± 0.3	50.5 ± 0.4	45.3 ± 0.6
DeepLabv3+ [13]	Xception	52.2	83.4	78.9 ± 0.2	92.9 ± 0.3	52.0 ± 0.5	47.4 ± 0.6
Segformer [38]	MiT-B2	26.1	56.8	79.3 ± 0.3	93.8 ± 0.4	57.9 ± 0.4	53.4 ± 0.4
Segmenter [39]	ViT-S/16	24.8	25.9	77.4 ± 0.4	91.6 ± 0.4	55.4 ± 0.5	51.5 ± 0.6
SAM2-UNet [40]	SAM2.1-Hiera-S	36.6	48.1	79.3 ± 0.3	92.9 ± 0.3	70.2 ± 0.3	61.8 ± 0.4
DINOv3-S [1]	ViT-S/16	**20.6**	**16.8**	75.4 ± 0.2	89.2 ± 0.3	69.8 ± 0.3	61.1 ± 0.4
LapDINO	ViT-S/16	23.6	20.1	**81.6** ± **0.2**	**95.9** ± **0.3**	**73.1** ± **0.3**	**64.3** ± **0.4**

**Table 4:** Per-class IoU comparison of different methods on the VOCD dataset. LapDINO achieves the highest IoU across all terrain categories, demonstrating superior semantic–geometric fusion for diverse off-road surfaces.

Method	Pavement	Unpaved	Snow	Water	Grass	Mud	Background	Mean
UNet	80.8	74.8	73.2	77.5	81.6	68.6	93.0	78.5
PSPNet	81.5	75.4	73.5	77.8	82.1	68.8	93.2	78.9
DeepLabv3+	81.4	75.1	73.6	77.6	81.9	69.4	93.2	78.9
Segformer	81.9	75.7	74.2	78.0	82.4	69.4	93.5	79.3
Segmenter	79.9	73.3	71.6	76.6	80.6	67.3	92.5	77.4
SAM2-UNet	81.9	75.6	73.7	77.9	82.3	70.1	93.6	79.3
DINOv3-S	78.3	71.7	69.5	75.1	78.4	63.8	91.0	75.4
LapDINO	**84.5**	**78.2**	**77.8**	**81.3**	**83.1**	**71.3**	**95.0**	**81.6**

**Table 5:** The effect of each module on performance.

No.	Adapters	LPE	MBA	BCF	mIoU ↑
1	×	×	×	×	75.43
2	✓	×	×	×	77.95
3	×	✓	×	×	78.73
4	×	✓	✓	×	79.24
5	×	✓	✓	✓	80.15
6	✓	✓	✓	✓	**81.57**

**Table 6:** Sensitivity analysis of adapter bottleneck dimension *r*. The baseline (No Adapter) achieves 80.15% mIoU. While smaller *r* yields higher accuracy, the marginal gain diminishes rapidly with increased parameter overhead.

No.	*r*	ΔParams (M)	ΔGFLOPs	mIoU (%)	ΔmIoU (%)
1	No Adapter	0	0	80.15	0
2	r=128	+0.03	+0.03	81.14	+0.99
3	r=64	+0.06	+0.05	81.57	+1.42
4	r=32	+0.12	+0.11	81.71	+1.56
5	r=16	+0.23	+0.21	81.89	+1.74
6	r=8	+0.46	+0.42	82.08	+1.93

## Data Availability

Data is available upon reasonable request to the corresponding author.
